# “Dreaming Undreamt Dreams” in Psychological Counseling with Italian Women Who Experienced Intimate Partner Violence: A Phenomenological-Interpretative Analysis of the Psychologists’ Experience

**DOI:** 10.3390/ijerph17176286

**Published:** 2020-08-28

**Authors:** Giorgia Margherita, Gina Troisi, Maria Ilaria Incitti

**Affiliations:** Department of Humanities, University of Naples Federico II, Porta di Massa 1, 80133 Naples, Italy; margheri@unina.it (G.M.); mariailariaincitti@hotmail.it (M.I.I.)

**Keywords:** intimate partner violence, trauma, post-traumatic dreaming, dreaming, psychological counseling

## Abstract

In psychological consultations with women who survive Intimate Partner Violence, it is essential to work on elaboration of the trauma as a complex trauma within the context of a relationship. We consider dreams to be a symbolic-representative process, which requires the right psychic, relational and contextual conditions to occur, and that is hindered when trauma is present. The objective of the present study is to investigate the meanings that psychologists working at anti-violence centers attribute to the clinical intervention with women victims of IPV, with a focus on the area of sleep and dreaming in a traumatic experience, and in the clinical work on the trauma. Twelve female psychologists were interviewed using the Interpretative Phenomenological Analysis methodology. From the analysis of the interviews, three main themes emerged: (1) *Day and night, neither awake nor asleep*, (2) *Anti Violence Centers: setting as a container of emotion?* and (3) *dreaming undreamt dreams.* The study highlights the importance of dreams as an indicator not only of psychic and mental functioning but also of the psychological relationship within a specific context.

## 1. Introduction

According to the World Health Organization [1], violence against women is a major public health problem and violation of women’s human rights in the world, and Intimate Partner Violence (IPV) is the most widespread form of violence. IPV is any form of concrete or threatened physical, sexual, psychological, or economic violence, or stalking, perpetrated by an ex or current partner. Within the Member States of the European Union, 22% of women have suffered physical and/or sexual violence at the hands of their partner, with specific country rates ranging from 13% to 32% [2].

In Italy, where our study was conducted, a national survey from the National Statistics Institute [3] revealed that 31.5% of women between the ages of 16 and 70 had experienced at least one episode of sexual or physical violence from a partner or ex-partner.

Violence against women has serious consequences for women’s psychophysical, sexual and reproductive health, especially in cases of IPV [1]. Several studies, in fact, indicate that women survivors of IPV tend to suffer from depression, panic attacks, suicide attempts, non-suicidal self-harm, alcohol or drug abuse as a consequence [4,5,6,7,8,9,10], and especially from post-traumatic stress disorder (PTSD) [11,12]. The trauma experienced by women post-IPV is different from the trauma that results from other stressful life events.

Post IPV trauma is, in fact, an interpersonal trauma, caused by another person, a man-made trauma [13,14] that is repeated, lasting and “complex” [15], with specific consequences on the emotional level [16,17,18,19]. The trauma makes the signification process difficult, which often happens with relational trauma because it destroys trust in a symbolically shared world [20].

The literature on PTSD has investigated the central role of sleep disorders [21,22,23,24,25] as a symptom linked to the state of hyperactivation, as well as the presence of post-traumatic nightmares, which are seen as intrusive symptoms related to the traumatic event [21,26,27,28,29,30,31,32].

Research into sleep disorders in women who are victims of IPV confirms that, apart from insomnia [10,33,34,35], many experienced difficulties in getting to sleep, tended to wake up very early and had disturbing dreams [28,36].

If the literature on post-traumatic disorders in IPV survivors highlights the significance of sleep disorders [10,28,34,35], less space has been given to the role of dreams. This study aims to try and fill this gap.

Studies that have investigated the characteristics of dreams post trauma have shown that traumatized individuals have more dreams, like threat simulations [36], and a higher frequency of high dream recall [37]. As regards the function that dreams have in relation to the trauma, some authors differentiate between the dreams that replicate the trauma, and nightmares, which they term sleep disorders, and those dreams that, on the other hand, contribute to the elaboration of the traumatic memory [26,38].

The importance of dreaming as a mental activity that regulates, integrates and repairs psychic processes is highlighted in the rich inter-disciplinary and transversal dialogue between models that range from cognitivism to psychoanalysis and to neuroscience [39,40,41,42].

From a psychodynamic point of view, which is our theoretical framework, we see dreaming as a symbolic-representative activity that gives meaning to the emotional experience. This is prevented by the traumatic experience, because it attacks the inner mental space where experience is stored, contained and elaborated [26].

Freud [43,44] already illustrated the nature of traumatic dreams, which could not be considered the expression of unconscious desire but rather an attempt to dominate the traumatic event and make it representable to the psyche.

Contemporary literature in particular shows how post-trauma dreams enable connections between the traumatic event and the associated emotions [31,45].

Diverse studies have shown that extreme traumatic experiences impact cognition, affect and the body, which indicates that treatment of post-trauma syndromes calls for an integrated approach based on the concept of mindedness, or a process of mentalization [46,47].

Clinical work on dreams following trauma, therefore, allows for the restoration of symbolic activity [40,48], the narrative reconstruction of the traumatic event and the repositioning of the traumatized subject [26].

Thus, dreams can serve as a useful indicator of the effectiveness of clinical work with traumatized patients: various studies interpret the presence of dreams with strongly emotional content to be a sign of psychotherapeutic change [42,49].

### 1.1. Working on Trauma in Services for Women Who Experienced IPV: The Function of the Operator’s Mind

Herman-Lewis [15] points out how, in therapy work with women who have suffered IPV, recovery from the trauma goes through various stages: construction of safe conditions, elaboration of the grief to transform the memory of the trauma and the rebuilding of social relationships. Although many studies focus on the consequences that IPV has on the psychophysical health of those who experience violence, few studies have focused on different types of clinical intervention and their effectiveness [50,51,52,53,54,55].

Diverse factors are at play: the intimate nature of the phenomenon, the ethical and political objections raised by feminist movements who see psychological counseling for women who experienced IPV as a way of pathologizing the victim and the fact that anti-violence centers’ (AVC) (These are centers that offer legal advice, free psychological support and sometimes even a refuge for women who need protection. The centers are financed and supported by Local Administrations, sometimes discontinuously: they are in fact often organized on a voluntary basis due to the absence of adequate and protracted economic funding over time. According to data provided by ISTAT [56], around 4400 operators worked in anti-violence centers in 2017 and 56.1% carried out this service on a voluntary basis.) interventions often focus on the emergency, offering women concrete help and protection [52]. Furthermore, as evidenced by the studies [54,57], the psychological processes that keep women in the violent relationship are ignored, as they are in IPV screening, and this impacts the way the interventions are structured.

Different countries provide different types of services for people who seek help as a result of IPV, but what emerges is a need to work on post-traumatic symptoms, on depressive aspects [58], on the risk of recurrence [59] and also on strengthening existing resources and social support [60].

Many prefer to use group intervention [61,62,63,64,65,66,67,68], leveraging the important therapeutic factors associated with groups like mirroring [63] and gender-based support. Recent studies have highlighted how evidence-based trauma-focused treatments such as prolonged exposure and cognitive processing therapy can help women who experience IPV [64].

Several studies have identified the importance of the use of narration with traumatized individuals [61,62] or those who suffered violence “at human hands” [68,69].

With women who have suffered violence, narrative therapy [70] proves very useful for the reconstruction of their story, and for bringing out emotional content in order to give meaning to their experience.

From a psychodynamic perspective, the clinical intervention itself is a form of narrative that helps to reconstruct the overall experience and contributes to the process of elaboration of the trauma by facilitating the reconstruction of the sense of the experience.

In our view, it becomes possible to symbolize the experience of the reality and the trauma because the work of another mind, that of the psychologist, prevents emotional flooding and digests the raw data of the sensory experience [71], transforming it into something that can be named, an essential prerequisite of the narrative process.

Therefore, the therapeutic “narrative” dimension, and the process of attribution of meaning within a relational framework that identifies the other as a witness, are essential elements for the treatment of severely traumatized persons [13,14].

These aspects have strong implications for the psychologists who establish contact with the area of trauma, and who need to revive thought processes. In fact, the literature emphasizes how working with traumatized subjects poses many problems for the psychologists themselves, and how it affects them as a person. The humanity of the therapist is the focal point of their engagement with the patient and a basic requisite for working with trauma.

Psychologists who work to provide support for victims of sexual abuse experience strong emotions, such as anger, frustration, pain, shock and horror, and they report flashbacks of experience and intrusive dreams and thoughts, as well as fatigue and patterns of disturbed sleep [66]. In particular, working with victims of IPV seems to profoundly influence the identity sphere and relationships [72,73,74,75,76].

Several authors [77,78] have proposed the terms *vicarious trauma* and *secondary traumatic stress* to describe the effects that working with trauma victims have on psychologists. Indeed, it has been shown that therapists working with female victims of violence experience a feeling of vulnerability that can manifest itself through fear of experiencing family violence, suspicious behavior or excessive control [79].

Starting out from a psychodynamic perspective, which shows how trauma impacts on thought processes and on mental functioning, obstructing symbolic and representation activity and damaging the subject’s psychic coherence [80], our study aims to fill a gap in the literature. Our work explores the mental activity of dreaming in IPV as there are currently no specific studies focusing on dreams within the clinical experience of women who suffer violence.

The quality and nature of dreams under traumatic conditions are explored in intrapsychic and relational terms, with particular attention to the possible conditions in which the dreams present, given that dreams, in a clinical context, require specific relational and contextual conditions in order to manifest themselves.

### 1.2. Objective

The present study aims at investigating the meanings that psychologists in anti-violence centers attribute to the clinical intervention with women who experienced IPV, with a focus on the area of dream and dream processes. Specifically, we decided to interview psychologists to study the subjective experience of those who work with traumatized users, using the sleep area and the dream function as an indicator of symbolic-representative activity. In this way, the study developed on two levels: on the one hand, it looked at the figure of the psychologist in her role as an observer and witness of the experiences reported by women and her investigation of post-traumatic symptoms relating to sleep and dreams; on the other hand, it explored the experience of the psychologist in her clinical relationship with the women, focusing on the importance attributed to sleep and dreams in their work.

## 2. Materials and Methods

This study proposes a qualitative research methodology using the interpretative-phenomenological analysis (IPA) approach [81]. The IPA approach is a particularly useful research method for exploring new study phenomena and very subjective themes that, at the same time, affect the social context. The IPA has an *idiographic* focus on how the participant gives meaning to his/her own experience, *hermeneutic* because the researcher’s interpretation of the participant’s meanings is important, and *phenomenological* because it gives importance to narrative truth and considers the participants the true experts of the phenomenon that forms the subject of the research. The objective of IPA is the analysis of specific experiences rather than the generalizability of the results in order to highlight unique perspectives as well as shared experiences, and consequently, small samples are commonly advocated for IPA studies.

This methodology allows a recursive and bottom-up (inductive) research process, identifying the processes of meaning that people use to give sense to their experiences.

### 2.1. Interview

In line with the aims of the study, an in depth and semi-structured interview was developed to try and explore the following areas:

1. Characteristics and meanings that psychologists in the anti-violence centers attribute to the psychological intervention with women who suffer IPV (*What is the structure of the intervention? What are the strengths and difficulties of the professional experience? Who are the figures involved? What is the role of the psychologist? What is the duration of the psychological intervention?*).
2. Emergence of dreams and sleep disorders as well as roles and functions attributed by the psychologist to the dream process during the psychological intervention (*Do women report suffering from insomnia? Do women report difficulty falling asleep or intermittent awakenings? Do women report post-traumatic dreams and if yes, what characterizes them? If dreams emerge during clinical support intervention, how are they used? If narration of dreams emerges, when does this usually happen? What meaning do you attribute to dreams in your clinical practice?*).

### 2.2. Setting and Procedure

The study was conducted in Campania, a region in southern Italy. In Italy, women who have experienced IPV are mainly taken care of in anti-violence centers (AVCs), managed by Non-Governmental Organizations, which is why we chose anti-violence centers as the context for our study.

Ten AVCs in the Campania region were identified and contacted by telephone or e-mail. Only the AVCs that offered psychological support to their users were included in the study. The 10 AVCs contacted all offered this service, but only 7 accepted to participate in the study, putting us in contact with 13 Psychologists. 12 of them accepted to participate in the study.

The interviews, lasting an average of thirty minutes, were recorded and transcribed verbatim so they could be processed with the IPA methodology. The interviews were conducted in the period between December 2017 and June 2018.

### 2.3. Participants

The participants, for whom we will use pseudonyms, compiled a form with age, psychotherapeutic approach, psychological intervention provided by the AVC and years of professional experience with women who have suffered violence (Table 1). At the time of the interviews, the participants were aged between 30 and 41 (Mean: 34.3, Standard Deviation 3.91). Clinical training covers different approaches. On average, the psychologists presented with about 8.6 years of experience in the anti-violence sector (M = 8.6, SD: 6.57). Clinical training covers different approaches, with a majority of psychologists with a systemic orientation (5), followed by operators with a psychodynamic orientation (3), cognitive-behavioral (2) and a minority with a bioenergetic orientation (1) and transactional analysis and Gestalt therapy (1).

On average, psychological intervention in the anti-violence centers involves 8 sessions.

All aspects of the study received informed consent from each participant, according to the ethical guidelines of the Helsinki Declaration. Participants were informed of the confidentiality of their responses and the anonymous treatment of their data. The protocol was approved by the ethics committee of the Section of Psychology and Educational Sciences, University of Naples Federico II, Italy.

### 2.4. Data Analysis

The narratives produced by the psychologists were transcribed and analyzed using the IPA methodology, which involves several phases, starting with a thorough and careful reading in order to become familiar with the interviews. Initially, we paraphrased the interview contents and made preliminary comments, then we extrapolated the emerging themes, before finally separating them into superordinate themes and sub-themes. Every phase of the analysis conformed to the standards of the IPA quality evaluation guide, such as member-checking and data-triangulation [82].

During each stage of the analysis, we cross-referenced the IPA method with our own psychodynamic theoretical perspective, which enabled us to identify hidden meanings within the text. The different stages of the analysis were cross-checked by the research group at various intervals, and no major discrepancies in the codification of content between researchers emerged. The emerging themes were then inserted into the table (Table 2) to compare them and then categorize them under superordinate themes. During this process, some themes were abandoned, because they overlapped with themes already present.

## 3. Results

The analysis led to the development of three superordinate themes that include several emerging themes present in the interviews, and in Table 2, their recurrence amongst the participants is also reported.

### 3.1. Day and Night, Neither Awake nor Asleep

The psychologists we interviewed often referred to the night as a fragile, precarious space, which is vulnerable to invasion from the partner’s violence, which can unexpectedly, and at any time, assail the victim, even after separation, through a state of constant terror or sudden flashbacks (*2a: The terror of the partner besieges the space of the night*).

“Our users have great difficulty in falling asleep, because they are really afraid of being close to this man, for the things he might do to her, for what they can suffer, and, if there are children, for the fear that this man could assault their children” (Arianna).

“The women often, even after they are separated from their partner, have difficulty falling to sleep because they still feel the need to stay awake and keep watch because in the past it was dangerous for them to fall asleep because the violence often happened at night. I can’t help thinking of one woman in particular who was woken up by the smell of burning and realized that her husband was trying to set fire to the bed she was sleeping in” (Sibilla).

In this context, the state of anxiety and hyperactivation due to trauma appears to be what remains even when the risk has gone, and this causes insomnia and intermittent sleep. The psychologists report that the trauma seems to induce a state of constant vigilance in the women (*2b: Impossible to abandon and impossible to regress*).

“These women do not talk about their own nightmares, but they tell of their state of anxiety, which makes them wake up…Their sleep is restless, sometimes they do not even remember their nightmare. They often say, ‘I woke up because I was afraid and anxious’” (Rosa).

“There are women who talk about not being able to sleep at all, about being insomniac ever since they were in the violent relationship and that they have got used to not sleeping even though the relationship is over” (Mafalda).

This state does not allow them to be completely asleep, nor really awake and lucid; therefore, there is no contact that allows them to make decisions and to listen to themselves, either with their eyes open or closed (*2c: What state of consciousness*). The psychologists observe that the sleep disorders, combined with the intermediate condition between sleep and waking that these women frequently experience, further hinders the processing of the trauma, and slows down an authentic process of contact with their real self. In the absence of an authentic waking state, the present and the future appear as extremely compromised temporal dimensions.

“Emotionally, the sleep disorder, in my opinion, increases their precarious level of disorganization, even mental, even more...our users never turn off their brains, they think, think, think, always about the same things. I noticed that the women are not lucid, and they seem to sleep when they are awake and when they sleep they are “awake”. They are never really in touch with themselves…” (Sibilla).

“The women live in a strange state somewhere between being asleep and awake. They always seem a bit dazed, maybe because they are sleep-deprived or maybe because their mind is stuck on those episodes of violence” (Diana).

### 3.2. Anti-Violence Centers: Setting as A Container of Emotion

Dreams do not seem to find much space within the counseling sessions with users of the anti-violence centers. Psychologists report the need to work on the concrete aspects dictated by the emergency in which women find themselves (*1a: The concrete in the foreground: the work on the emergency*). The psychological work happens mainly at the level of the here and now reported by women users: risk assessment, recognition of violence, development of autonomy.

“So, in my opinion, in this type of emergency-centered pathway, there is not enough time to devote to an in-depth study that is a little more specific regarding nightmares, these traumatic experiences which, however, concern other spheres. Here, first of all, we must make sure that the users are safe” (Maya).

“Our work is heavily influenced by the pressure imposed by an outside world where terror reigns supreme, we are constantly in touch with the risk these women are exposed to and this makes it difficult to work on deeper aspects” (Arianna).

In AVCs, the lack of resources necessary for a more in-depth and continuous clinical intervention also explains why the focus is on concrete aspects. All the operators complain about the difficult and precarious conditions in which the AVCs operate, which means they need to work as quickly and efficiently as possible. This leads to a great sense of frustration (*1b: The impotence of the psychologist*).

“We (psychologists) are forced to fix appointments differently, sometimes our counseling sessions overlap; while I have a psychological counseling session, the user outside is even forced to wait 20 min because the lawyer was using the room before for legal advice. Since our AVC has no more funding, we decided to reserve psychological counseling for our most serious users and so our work is very limited” (Eva).

“The eight-interview limit makes the intervention fall somewhere between impotence and omnipotence. We have to do everything we can, in the shortest time possible, and this makes us feel that we don’t have the tools we need to achieve anything while feeling it is our responsibility to do something” (Arianna).

Many psychologists highlight the importance of teamwork, which compensates for the lack of resources at the AVC, making the intervention more complex and targeted. The comparison with other kinds of psychologists, the group discussions and the communication with the social services, make it possible to take care of the women on multiple fronts, thus reinforcing the work of the individual psychologist (*1c*: *A female embrace: limit and resource*). Many psychologists underline the importance of a meeting between women that can allow greater acceptance and understanding of the dynamics involved, but they also underline how this can become a limit when identification mechanisms come into play that can hinder the intervention because they are not thought through via group supervision.

“In anti-violence centers, it is important to work in a group where each professional (the lawyer, the caseworker, the psychologist, etc.) does their own part. To describe our way of working, we use the metaphor of an embrace, and the women, at the end of their treatment, report feeling embraced in a hug by all of us. That embrace is important also for us because the weight, the thoughts and responsibilities you feel are too many if you don’t share them” (Sibilla).

“The fact that we are all women makes it easier to understand each other, to recognize yourself in the other, but sometimes it also gives rise to a sense of competition which you have to consider in the relationship” (Alda).

### 3.3. Dreaming Undreamt Dreams

All psychologists report that dreams do not appear in the clinical work with these women. There are various reasons for their absence (*3a*: *For women, it is impossible to dream*). First of all, in AVCs, during the psychological counseling sessions, women tend not to bring their dreams and discuss them.

“About dreams? No, users in the AVC do not usually discuss their dreams with us ... it is not a dimension that finds space here. The dream is a very faraway dimension for them” (Arianna).

“Women do not tell us their dreams, it is very difficult, the feeling I have is that they don’t dream precisely because they have such problems in sleeping” (Barbara).

The sporadic appearance of the dream has the characteristics of a hallucination, a sort of image that is not inserted in a narrative thread and that strongly repeats the scene of violence (*3b: The image from which to escape*).

“In the sessions, women tend to report episodes in which they are physically and psychologically threatened…usually they have nightmares related to death. They experience the assaults in their dreams” (Mafalda).

“When nightmares occur, they are like flashbacks to what the women experienced in the violent relationship. I remember a woman who said she used to wake up because she had the impression someone was always about to get her” (Emma).

However, despite the absence of any real clinical work on dreams, it is common for the psychologists “to dream” about their users at night and during the day (*3c: The vicarious function of the mind of the other. I am the one who dreams of the women I meet*).

“It’s happened to me, and to some of the rest of us, that we dream about the people who use the center. I usually dream about them, I dream about all the things they discuss with me, as if there was a need for me to digest their stories…if their stories or something they told me makes me worry or left me upset, I dream of it, as if I needed to keep thinking about it” (Sabina).

“They hardly ever talk about their dreams with me but, strangely, I often dream about the women I’m working with. Dreams where what happened to the women is all mixed up with other cases. I thought this might be because it stirred up personal memories or because it was something my head needed…” (Maya).

“I dream about the women I meet because they get into my head, and when I dream about them, I understand more about what the woman has made me think about and I think it’s useful for our clinical relationship. It’s almost as if I dream instead of them” (Barbara).

## 4. Discussion

The results highlight three interpretative areas. The first is the way psychologists report that the mental functioning of women who have suffered violence is still under siege, even after the violent relationship has ended. The presence of sleep disorders, according to the literature [28,33,34,83], shows that the women cannot allow themselves to rest, and that the chance to dream is hindered, since the women are very much anchored to the concrete and practical.

In the representations of the psychologists, these women’s nights are marked by insomnia and intermittent sleep [10,84], due to the feeling of uncertainty and the possibility that the partner’s violence may occur even at night [85], causing frequent awakenings and giving a feeling of unpredictability and intermittence that prevent the women from sleeping. The psychologists describe a particular state of consciousness that characterizes women, a state of perennial vigilance that, despite their extreme tiredness, does not allow them to sleep, or to be really awake and lucid, in a kind of *anesthesia of the conscience* [86,87].

The second area is represented by the psychologist’s feelings of impotence, because they believe that their intervention is not enough, neither in terms of the women and what happened to them, nor in terms of the resources available in the center. In the consultation space available at the anti-violence centers, it seems that dreaming does not come up, regardless of the approach and theoretical orientation of the participants. The daily commitment of the psychologists, often volunteers, is mainly geared towards encouraging awareness of the violence, in risk assessment [59], in the exploration of post-traumatic affects [16] and in providing the women with the psychological and economic tools they need to escape the situation of violence. In this context, team-work seems to be an essential resource if the necessary space for thought is to be preserved.

The third level is represented by the clinical relationship between psychologists and women victims of violence, in which there is a clear connection between the women’s capability to dream and the psychologist’s ability to elaborate traumatic content. In this relationship, the restoration of the ability to dream seems to be transmitted by the psychologist’s ability to dream. In clinical work with this type of user, the absence of dreams is not a marginal aspect of the research and seems mainly due to the collapse of representative activity following the trauma. The experience of violence also comes back to the women through recurring dreams, which reconfirms what is already reported in the literature [28,35].

What seems to fill these women’s desolate dream world and, albeit rarely, makes its presence felt during the psychological counseling work, is the aggression they experienced, and the threats and even the image of their own death. These take on the character of a violent evacuation of split psychic contents; in fact, traumatic memories post-trauma often present intrusively as affective states—as sensory fragments with no verbal or visual representation [88]. Faced with the user’s lack of unity and internal narrative coherence, the psychologists’ dreams take over. In fact, in at least half of the cases, the psychological counselors emerge as the true dreamers in the research. It seems that the collapse of the users’ ability to think, and the overload of violent material, means that it is the psychologists who deal with the unelaborated material [71,89] and the interrupted or “not dreamed” dreams [90] of users. In the interviews, the meaning that the psychologists give their dreams is that of a constant thought, determined by their worrying about the women, and by the strong emotional content in the stories they hear. It seems that, through their dreams, it is possible to use the contents proposed by the user and, at the same time, to perform for users a function of reverie [71], offering a vicarious mind, in the absence of material they actually “dreamed of”.

The findings of our work have some clinical and research implications. A focus on dreams could be a useful aspect for clinicians working with women who have undergone violence to investigate, regardless of their approach, because it could provide diagnostic pointers regarding mental function in relation to the process of elaboration of the trauma. This is in line with a broad range of literature that shows, in other contexts, the impact that trauma has on dreaming and thought processes [42].

The emergence of dreams as narration, connected to processes of symbolization, is different from the experience of certain nightmares which feature mental experiences that remain concrete, unconscious and unobservable for the subject, and are the product of a mind which needs to evacuate itself by getting rid of emotion.

Dream activity as elaboration, which proved absent in the women, emerged in the clinical relationship, via the psychologists. Just as *vicarious trauma* describes the conditions that endanger the psychological well-being of operators who deal with trauma victims, our study revealed a process of “*vicarious dreaming*” whose function was one of elaboration and support.

Our results also confirmed the need to look at the trauma experienced by women post IPV in a different way from the traumas that occur as a result of other stressful life events [91]. Working with post-IPV trauma as interpersonal trauma implies that particular attention needs to be paid to the relational dimensions of the intervention.

The study shows how the traumatic experience of violence impacts on some areas of mental functioning and on the quality of bonds and relationships, both for the women who suffer it, and for the operators who take charge of it.

The psychodynamic lens that takes into consideration the global clinical and therapeutic framework has highlighted the quality of primitive affective and emotional dimensions that cannot be represented, that cannot be “dreamed” and that manifest themselves through unconscious transference–countertransference dynamics, which involve health care providers, like clinical psychologists. This highlights the needs for specific improvements regarding both policy and provision of Health Services.

## 5. Conclusions

The present study offered some interesting insights into the characteristics of the psychological intervention in services for women who suffer IPV and the roles that dreams and the dream process play for psychologists. Our data regarding sleep shows how hard it is for psychologists to pay much attention to dreams in the context of their work at the anti-violence centers. This is because they are dealing with emergencies, because of the timing of the sessions and because funding is so precarious it is impossible to offer their users continuity. In our opinion, this also reflects that, on a societal level, even in today’s world, gender violence often remains “invisible”.

Although it emerged from the psychologists’ narratives that there is no clinical work on dreams, dreams found their own space within the psychologist’s mind. The psychologist seems to act as a kind of supporting Ego by dreaming on behalf of the woman and to activate a process of elaboration of the experience which the woman is unable to do because the impact of the trauma is too strong. The ability to restore a symbolic thread and a connection between fragmented temporal dimensions cannot therefore be separated from the witness function that psychologists offer in the clinical relationship [14].

As the psychologists often pointed out, a psychological space needs to be created within the anti-violence centers, which “contains” and creates the presuppositions to encourage processes of elaboration of the traumatic material. In this sense, spaces like team-work and supervisions could use group work to leverage transformation. The group, in fact, appears to be a privileged setting for the elaboration of traumatic aspects, it works as a transformative tool [71], offering through the other members elements which seem unavailable to the individual’s mind [92].

This study presents some limitations. Although we did not carry out any comparative analysis of the clinical approaches used by participants, we did not identify any significant variation in the clinical orientation reported. Furthermore, it would be interesting to consider the possible evolution of dream processes over a longer course of treatment with women who have suffered violence, and where the intervention is less emergency-focused than it tends to be in the anti-violence centers. Moreover, it would be interesting to make a comparison with other traumatic situations to understand if the specificity of this clinical relationship, the fact that it is all-female and there is a greater tendency to identify with the user, tends to increase the psychologists’ dream activity. It would also be interesting to evaluate to what extent the psychologists’ dream activity is a factor for supporting and transforming the clinical relationship with women who have suffered violence. In such terms, the literature [93] shows that dreaming about patients not only helps therapists to understand themselves better, but also offers important insights into the dynamics of the clinical relationship. Therefore, dreaming could act not only as an indicator of psychic functioning and provide useful diagnostic elements, but also as an indicator of the clinical relationship. Our work focused on the work of the psychologists, but future studies should interview women who have suffered violence, exploring their dreaming activities through the administration of specific tools [94,95,96,97], like the Mannheim Dream Questionnaire (MADRE) that measures various aspects of dreaming [98,99].

This study proposed an original perspective for monitoring the therapeutic process in anti-violence centers and takes dreaming as an indicator of how the elaboration of the trauma and the clinical relationship is going.

An original dimension of the study was the ability to bring together a qualitative research method and psychodynamic theory, in line with previous studies [100,101,102] that consider interpretative phenomenological analysis an approach that offers suitable material for psychodynamic understanding and interpretation. Qualitative research results, such as IPA, should be considered in terms of vertical generalizability [103], interested in building interpretative theory, expanding knowledge and contributing to existing theories and the generation of new hypotheses, proposing a reassessment of what has been considered known or understood about the investigated phenomenon, differently from the horizontal generalizability which points towards results applicable in all contexts.

In conclusion, this work offered a series of reflections that could be used to inform and improve the psychological counseling that is provided as part of the support services for women who have experienced violence and highlights how complex psychological work is in terms of emotional involvement.

## Figures and Tables

**Table 1:** Participants.

Pseudonym	Age	Clinical Approach and Orientation	Experience in Anti-Violence Centers	Counseling Cycles
Alda	32	Training in Psychodynamic Psychotherapy	2 years and 6 months	Cycles of 8 sessions
Rosa	30	Training in Systemic Therapy	2 years	Cycles of 8 sessions
Sibilla	31	Training in Systemic Therapy	4 years	Cycles of 8 sessions
Maya	41	Training in Systemic Therapy	10 years	Cycles of 8 sessions
Arianna	38	Training in Systemic Therapy	13 years	Cycles of 4 sessions
Sabina	31	Training in Psychodynamic Therapy	5 years	Cycles of 8 sessions
Emma	35	Training in Transactional Analysis and Gestalt Therapy	5 years	Cycles of 8 sessions
Mafalda	36	Training in Cognitive-Behavioral Therapy	10 years	Cycles of 12 sessions
Barbara	41	Training in Psychodynamic Therapy	15 years	Cycles of 5 sessions
Melania	31	Training in Bioenergetic Analysis Therapy	4 years	Cycles of 5 sessions
Diana	34	Training in Systemic Therapy	7 years	Cycles of 5 sessions
Eva	31	Training in Cognitive-Behavioral Therapy	4 years	Cycles of 5 sessions

**Table 2:** Superordinate and subordinate themes.

Superordinate and Emerging Themes	Alda	Rosa	Sibilla	Maya	Arianna	Sabina	Emma	Mafalda	Barbara	Melania	Diana	Eva
**Day and night, neither awake nor asleep**												
The terror of the partner besieges the space of the night	*	*	*		*		*			*		*
Impossible to abandon and impossible to regress	*	*	*	*	*	*	*	*	*	*	*	
What state of consciousness	*	*	*	*		*	*	*			*	
**Anti-violence centers: Setting as a containers of emotion**												
The concrete in the foreground: the work on the emergency		*	*	*	*		*		*	*		*
The impotence of the psychologist	*	*	*	*	*			*			*	*
A female embrace: limit and resource	*	*	*	*				*			*	
**Dreaming undreamt dreams**												
For women it is impossible to dream	*	*	*	*	*	*	*	*	*	*	*	*
The image from which to escape	*		*	*			*	*	*	*	*	*
The vicarious function of the mind of the other. I am the one who dreams of the women I meet		*	*			*	*		*		*	

* Indicates the frequency of the theme among participant.
